# Foreword to the CommDat special virtual issue on the raw diffraction data workshop and microsymposia at IUCr2023

**DOI:** 10.1107/S2414314625007096

**Published:** 2025-09-26

**Authors:** Loes M. J. Kroon-Batenburg

**Affiliations:** a Structural Biochemistry Bijvoet Centre for Biomolecular Research Utrecht University Universiteitsweg 99 3584 CG Utrecht The Netherlands

**Keywords:** CommDat, raw data, data reuse

## Abstract

Papers from the CommDat workshop on ‘Raw Diffraction Data Reuse: the Good, the Bad and the Challenging’ and microsymposia at the IUCr2023 Congress in Melbourne are presented.

At the 2023 IUCr Congress in Melbourne the Committee on Data (CommDat) organized a workshop ‘Raw Diffraction Data Reuse: the Good, the Bad and the Challenging’ (see https://www.iucr.org/resources/data/commdat/melbourne-workshop for the program, abstracts and presentation slides). The full-day workshop was aimed at: (i) discussing current practices in raw data archival and sharing, (ii) educating those who generate and deal in crystallographic data on best practices in data reuse in various categories of crystallographic science by leading experts, (iii) offering a summing up, including the role of IUCrData’s new Raw Data Letters. The floor was given to facility and raw data archive providers as well as to raw data reusers.

Papers arising from the contributions to this event as well as the CommDat-organized microsymposia A118 ‘Raw Diffraction Data Reuse: Warts and All’ and A119 ‘Interoperability of Crystallographic Data and Databases’ are collected in this special virtual issue.

The feature article by Helliwell *et al.* (2024 ▸) describes the efforts and the progress of the IUCr Diffraction Data Deposition Working Group, and its successor CommDat, from 2011 until today, to investigate the possibilities and benefits of archiving raw diffraction data for retrieval and reuse by other scientists. Several workshops have been organized during this period and a range of papers has been published. A notable outcome was the launch of Raw Data Letters, a section of *IUCrData*, an innovation by IUCr journals and the IUCr Committee on Data (CommDat) (Kroon-Batenburg *et al.*, 2022 ▸).

Many scientific communities are moving to Open Science policies, which is increasingly requested by funding agencies. Research data should be made public and should adhere to the FAIR principles (Wilkinson *et al.*, 2016 ▸). Open data support a cooperative data analysis, sharing data with other unrelated scientists who may re-analyse the data in order to extend understanding or even to make new original discoveries. The re-analysis of data is one of the principal aims of Raw Data Letters. Very revealing is the paper by Brink *et al.* (2024 ▸) who point out that the widest impact of our community’s effort can be achieved with interoperability between the various disciplines. They also note that FAIR data are not necessarily Open, *e.g.* the data could be behind a paywall but still adhere to the FAIR data principles.

In his keynote lecture, Andy Götz (2023 ▸) discussed the coordinated action of PaNOSC and ExPaNDS to increase raw data release to the public after a three year embargo period and facilitating the underpinning science publications with a DOI to the raw data archive. Concerted initiatives are taken to ensure all facility data are FAIR (Murphy *et al.*, 2025 ▸). The DAPHNE4NFDI consortium aims to improve metadata capture through consistent workflows at RIs supported by user-driven online logbooks that are linked to the data collection to achieve the common aim of FAIR open data.

Advances in instrumentation and detector technologies allows data collection with extremely high data rates and produces huge data volumes. Managing rapid data collections is made possible by efficient data processing pipe lines that process data on the fly (Karnevskiy *et al.*, 2024 ▸; Tolstikova,2023 ▸).

An important aspect that is raised in some papers is the carbon footprint related to large amounts of data storage. These days users of photon and neutron (PaN) RIs produce large data volumes of up to 1 PByte per day in some cases. This requires enormous capacities for data storage. Barty (2023 ▸) calculated that from 2028, PETRA IV will need 500 PB of disk storage to keep the data for 180 days and up to 1 EB of tape storage per year. This will cost ∼200 M Euro per year and consume 1–2 MW of power. Keeping all data for 10 years will be unaffordable. Do we have to make a choice of which data are worth storing? What are the rights and responsibility of the user? Although it is currently still technically feasible to save all raw detector output, with the increasing data rates this will become impossible. Bernstein & Jakoncic (2024 ▸) discuss the various lossless compression algorithms for different types of data. In particular, for serial X-ray crystallography (SX) one may have to consider not storing all data, *e.g.* store crystal hits only, store indexed frames only, use lossy compression methods, store data only when it yields results, store a random sample of the data. Ultimately, one has to consider lossy compression and the paper by Galchenkova *et al.* (2024 ▸) discusses this in detail. They conclude that for serial crystallography, lossless compression alone is of some use in reducing data volumes, but is highly dependent on the detector and experiment and is ultimately limited in the compression ratios that can be achieved. This paper is also an example of raw data re-use. They show that software improves over time and careful reprocessing of previously collected data can deliver much better results at a later point in time.

While archiving primary research data, *i.e.* raw diffraction images, is the ultimate Open Science goal, some researchers believe that archiving unmerged structure factors could be a good alternative. Currently, in the PDB and CSD merged structure factors are already part of the deposition and required by many journals. Unmerged reflection data are included in CIF files as an appendix containing individual recorded reflections with scan angle and frame number and/or *x*,*y* positions on the detector. Vonrhein *et al.* (2024 ▸) show the benefits of having and using the additional information in the CIF file, *e.g.* for removing reflections contaminated with ice scattering and diagnosing radiation damage.

A critical overview on data processing, archiving and about what raw data actually are in powder diffraction studies is given by Casati & Boldyreva (2025 ▸). It is a very valuable resource of information on types of data and data processing dependent on the purpose of research: qualitative and quantitative phase analysis, structure solution from a powder diffraction data study of crystallinity, disorder, particle size distribution, strain or stress and temperature of pressure-induced phase changes. They conclude that ‘access to the primary data can be a source of new knowledge even years after the data have been collected originally’. The paper of Kabekkodu & Blanton (2024 ▸) describes the efforts made by the International Centre for Diffraction Data (ICDD) in archiving powder X-ray diffraction raw data in the Powder Diffraction File (PDF) database, which as of 2025 contains 20800 raw data sets. Most data are published as XY ASCII data and it needs additional metadata, like instrument parameters, to make full (re)use of the data. They advocate that scientific journals should encourage authors to submit powder diffraction raw data as powder CIF (https://www.iucr.org/resources/cif/dictionaries/cif_pd) along with required metadata that will also be beneficial for machine learning research.

Minor (2023 ▸) talked about proteindiffraction.org, the most extensive archive of raw diffraction data in macromolecular crystallography. He brought forward the difference between data dump *versus* resource. Harvesting of metadata makes the difference and in such a way the data sets are validated when stored on this archive. A similar archive, SBGrid databank, which is an initiative of the SBGrid consortium, was discussed in microsymposium A104 by Sliz (Nicholson *et al.*, 2023 ▸). A very useful approach to verifying the correctness of metadata is carried out by auto (re)processing. A poster connected with microsymposium A118 by Helliwell *et al.* (2023 ▸) brings forward that the FAIR principles do not include data quality. Recently, PDBj launched the raw diffraction data archive XRDa, allowing the combined evaluation of raw data, processed structure factors and derived structural coordinates. A systematic analysis is made of data quality in a CODATA GOSC case study with the aim of arriving at single, definitive, protein models derived from their raw diffraction data sets. Reuse of processed diffraction data is exemplified in a paper on the *CheckMyMetal* server through which macromolecular structures in the Protein Data Bank (PDB), from X-ray crystallography and cryo-EM, can be validated by the characteristics of their metal-binding sites (Gucwa *et al.*, 2024 ▸).
